# Application Combining VMD and ResNet101 in Intelligent Diagnosis of Motor Faults

**DOI:** 10.3390/s21186065

**Published:** 2021-09-10

**Authors:** Shih-Lin Lin

**Affiliations:** Graduate Institute of Vehicle Engineering, National Changhua University of Education, No.1, Jin-De Road, Changhua City, Changhua County 50007, Taiwan; lin040@cc.ncue.edu.tw

**Keywords:** VMD—ResNet101, intelligent fault diagnosis, motor fault

## Abstract

Motor failure is one of the biggest problems in the safe and reliable operation of large mechanical equipment such as wind power equipment, electric vehicles, and computer numerical control machines. Fault diagnosis is a method to ensure the safe operation of motor equipment. This research proposes an automatic fault diagnosis system combined with variational mode decomposition (VMD) and residual neural network 101 (ResNet101). This method unifies the pre-analysis, feature extraction, and health status recognition of motor fault signals under one framework to realize end-to-end intelligent fault diagnosis. Research data are used to compare the performance of the three models through a data set released by the Federal University of Rio de Janeiro (UFRJ). VMD is a non-recursive adaptive signal decomposition method that is suitable for processing the vibration signals of motor equipment under variable working conditions. Applied to bearing fault diagnosis, high-dimensional fault features are extracted. Deep learning shows an absolute advantage in the field of fault diagnosis with its powerful feature extraction capabilities. ResNet101 is used to build a model of motor fault diagnosis. The method of using ResNet101 for image feature learning can extract features for each image block of the image and give full play to the advantages of deep learning to obtain accurate results. Through the three links of signal acquisition, feature extraction, and fault identification and prediction, a mechanical intelligent fault diagnosis system is established to identify the healthy or faulty state of a motor. The experimental results show that this method can accurately identify six common motor faults, and the prediction accuracy rate is 94%. Thus, this work provides a more effective method for motor fault diagnosis that has a wide range of application prospects in fault diagnosis engineering.

## 1. Introduction

An electric vehicle is essentially different from a traditional internal combustion engine vehicle. It mainly uses electrical energy to drive the motor through a drive control system to cause the vehicle’s wheels to rotate and the vehicle to travel. Electric vehicles have become an important new type of green transportation with the characteristics of being clean and environmentally friendly and providing the efficient use of multiple energy sources.

Therefore, the detection of electric vehicle motor faults is very important, and the faults may greatly affect the overall performance of an electric vehicle or cause accidents. Not only in electric vehicles, mechanical equipment vibration monitoring and fault diagnosis technology is widely used in large, high-speed rotating machinery in the electric power, petrochemical, metallurgical, and other industries. Modern industrial equipment and systems are becoming larger and more complex, and the reliability, availability, maintainability, and safety fault diagnosis of mechanical equipment has received more attention. To promote the research of researchers on the mechanism of mechanical equipment failure and diagnosis technology. There are many related research works on the detection of motor faults. The research project of Murphey et al. [1] was mainly to detect faults and find out the problems caused by the switches in the inverter. The research was based on the theoretical basis of the electric drive, and the researchers developed a simulation model to simulate the normal state with all single switches and faults after a short circuit for verification. The research results show that through the machine learning method, faults can be correctly classified in a wide range of operating areas. Kankar et al. [2] studied the use of artificial neural networks (ANN) and SVM for the fault diagnosis of ball bearings. The research employed a high-speed rotor test bench supported by rolling bearings. As a result, the vibration responses of various defects of the ball bearings were obtained. Tashakori and Ektesabi [3] proposed a simple fault diagnosis technology for electric vehicles to diagnose faults in the brushless DC motor drive of the wheel. The fault diagnosis algorithm proposed in the research does not require a great deal of calculation work, and the results show the correct detection and identification of the switching fault of the BLDC motor inverter. Praveenkumar et al. [4] proposed the application of machine learning technology for automobile gearbox fault diagnosis. In the experimental study, the vibration signals of the gearbox under good and faulty conditions were collected. Then, the statistical features from the vibration signal were extracted, and the SVM method was used for fault identification. Ulatowski and Bazzi [5] proposed a combinational logic method to identify faults in the powertrain of electric vehicles (EVs). The method proposed in the study obtained more than 20 different faults in different drive cycle times and with different transmission system components (motors, inverters, transmissions, and sensors). The results show that the method can robustly and successfully diagnose different faults. Vakharia et al. [6] proposed a method of using multi-scale displacement entropy as a feature selection tool for ball bearing fault diagnosis. The research results indicate that the feature extraction technology applied to multi-scale permutation entropy can obtain improved classification accuracy. Ma et al. [7] proposed the black-and-white box method for diagnosing and reducing the abnormal noise of the Hub Permanent Magnet Synchronous Motor (HPMSM). The method of the research results improves the diagnosis and optimization efficiency of HPMSM abnormal noise. Xu et al. [8] proposed the literature analysis of the fault mechanism and diagnosis technology of range-extended hybrid electric vehicles and discussed the development trend of the fault diagnosis technology, providing the theory for the practical application of the state monitoring and fault diagnosis of the range extender of electric vehicles. Zhou et al. [9] proposed a motor torque fault diagnosis method for four-wheeled independent electric vehicles using an unscented Kalman filter. In the research, the authors transformed the problem of motor fault diagnosis into a problem of fault parameter identification and realized the identification of the unscented Kalman filter. Qi et al. [10] proposed a machine learning technique applied to data analysis and fault diagnosis in a reciprocating compressor system. Ali et al. [11] proposed a practical fault diagnosis method based on machine learning in laboratory experiments. In this research, two signal processing techniques, matching pursuit and discrete wavelet transform, were selected for feature extraction. Three classification algorithms, SVM, K-nearest neighbor (KNN), and integration, and 17 different classifiers provided in the MATLAB Classification Learner Toolbox were used to evaluate the performance and applicability of different classifiers to induction motor fault diagnosis. Hu, C. et al. [12] proposed the analysis of abnormal noise and vibrations of hybrid vehicles in pure electric driving mode. The research first conducted frequency analysis to determine the source of noise and vibration and then employed a few measures to reduce noise and vibration levels. The experimental results show that the gear meshing in the compound planetary gear set was the main source of noise and vibration. Huang et al. [13] proposed the use of the original time signal and frequency spectrum to predict the abnormal sound identification and diagnosis method of shock absorbers based on the deep neural network (DNN). Huang et al. [14] proposed a current sensor fault detection method for a built-in permanent magnet synchronous motor torque closed-loop control system based on a sliding mode observer. Chang et al. [15] used a hybrid method to create a fault diagnosis status monitoring system for induction motors. The laboratory results showed that the health status of induction motors could be successfully diagnosed. Various damages can also be classified into stator faults, rotor faults, bearing faults, and eccentric faults. Huang et al. [16] developed an integrated fault diagnosis algorithm for the motor sensor of an electric vehicle independently driven by the wheel. The article presents an integrated high and low-level fault diagnosis method for the advanced fault diagnosis of vehicle dynamics. Jing et al. [17] studied an adaptive multi-sensor data fusion method based on a deep convolutional neural network for planetary gearbox fault diagnosis. The research results show that their method can effectively detect the condition of the planetary gearbox with the best diagnostic accuracy among all the comparison methods in the experiment. Hsueh et al. [18] studied a deep CNN (convolutional neural network) model to automatically extract robust features from gray-scale images to diagnose faults in induction motors. The experimental results show that the proposed method achieves good accuracy in the fault diagnosis of induction motors. Hsueh et al. [19] researched and proposed the sequential self-separation method (OSSM) method of rotation speed in fault detection and monitoring. The research conclusions show that the proposed method can effectively identify the leakage faults of in-wheel motors under different working conditions. Jing et al. [20] studied an adaptive multi-sensor data fusion method based on a deep convolutional neural network for planetary gearbox fault diagnosis. The research results show that their approach can effectively detect the condition of the planetary gearbox with the best diagnostic accuracy of all the comparison methods in the experiment. Goyal, D. et al. [21] proposed the design and development of a non-contact vibration sensor to obtain vibration data for bearing health monitoring under load and speed changes. Their research method used selected features, which were passed to SVM and the ANN to identify and further classify various bearing defects. He, C. et al. [22] proposed a complex system fault diagnosis method based on compound multi-scale weighted permutation entropy and machine learning. This research presents a new type of rolling bearing fault diagnosis method that combines extreme-point symmetric mode decomposition (ESMD), Composite Multi-Scale Weighted Permutation Entropy (CMWPE), and the Multiple Adaptive Constraint Strategy (MACGSA) Optimized Least Square SVM (LSSVM) Gravity Search Algorithm Method. Meckel et al. [23] proposed a system based on machine learning to establish an online diagnosis in hybrid electric vehicle models. Their research platform was a fault injection framework and data processing algorithm for active fault diagnosis and recovery evaluation. Chang et al. [24] studied the fault diagnosis of permanent magnet synchronous motor demagnetization with three states: normal, mild demagnetization failure, and severe demagnetization failure. The research conclusions show that this method can achieve 96% accuracy to reveal the demagnetization of PMSM. Gundewar and Kane [25] published papers summarizing the main faults of induction motors, the latest diagnostic methods and advanced signal processing technology, and the practical applications of electric vehicles. Xiao et al. [26] developed a fuzzy preference method based on multi-sensor data fusion technology in fault diagnosis. Their research proved the rationality and effectiveness of the scheme in conflict and fault diagnosis management. Tra et al. [27] proposed a study on diagnosing initial bearing defects with a convolutional neural network (CNN) trained by the random diagonal Levenberg–Marquardt (S-DLM) algorithm at variable operating speeds. Hua et al. [28] presented the latest developments in the noise, vibration, and harshness of pure electric vehicles. Rauber et al. [29] studied machine learning for the fault diagnosis of vibration signals. The research method was experimentally compared with four different classifiers: K-nearest neighbor, SVM, random forest, and a one-dimensional convolutional neural network. The study identified common methodological evaluation flaws of machine learning methods used for fault diagnosis. Toma et al. [30] studied the use of genetic algorithms (GAs) and machine learning models in bearing fault diagnosis. The study concluded that the accuracy of the three classifiers reached more than 97%. Xiao et al. [31] studied the use of unsupervised deep learning to maximize mutual information in motor fault diagnosis. The research results prove that this method was superior to many popular unsupervised and fully supervised learning methods. Xue et al. [32] studied the use of artificial hydrocarbon networks (AHNs) for intelligent diagnosis to detect the mechanical failures of in-wheel motors (IWM). The research developed a complex error function to optimize the information of the classification target and define the distance error ratio to evaluate the performance.

The fault diagnosis method based on vibration signal processing is currently widely used, known as empirical mode decomposition [33]. The data can be decomposed into the sum of a series of intrinsic mode functions (IMFs) from high frequency to low frequency. However, EMD is prone to the problems of mode mixing and end effects. With the rapid development of time-frequency analysis methods, Dragomiretskiy et al. [34] proposed a new signal multi-scale time-frequency analysis and processing method: variational mode decomposition (VMD). VMD is a non-recursive signal decomposition method that avoids the traditional EMD recursive component selection process. The signal decomposition process was completely carried out in the variational framework. Through the construction and solution of the constrained variational model, the signal was decoupled into several IMFs with limited bandwidth, and the signal was adaptively decomposed according to the frequency domain characteristics of the signal. Due to the advantages of VMD in the field of complex signal analysis, it has been well applied in mechanical fault extraction and signal component extraction. Artificial intelligence (AI) technology is widely used in mechanical failure prediction and health management (prognostic and health management, PHM). Deep learning algorithms such as CNN [35] and RNN [36] are good image classification methods. He [37] proposed residual networks 101 (ResNets101), the main idea of which is to add a direct connection channel to the network, allowing part of the output of the previous network layer to be retained, simplifying the network structure, and improving the accuracy of classification.

The general mechanical system fault diagnosis system is physically divided into five parts: mechanical measurement, monitoring and protection, data acquisition, vibration status analysis, and network data transmission. Functionally, the mechanical system condition monitoring and fault diagnosis system can be divided into three parts: data collection, condition monitoring, and fault diagnosis. At present, typical mechatronic products such as electric vehicles, CNC machine tools, and AC servo drives are developing in the direction of digitization, miniaturization, complexity, and high precision, presenting new challenges for fault monitoring and diagnosis. The development trend of artificial intelligence mainly concerns the use of artificial methods and technologies to imitate, extend, and expand human intelligence to allow machine intelligence diagnosis. The AI technology applied as mechanical fault diagnosis systems can traditionally be divided into three categories: expert systems [29], artificial neural networks [21], and fuzzy set theory [26]. Expert systems are mainly used for complex mechanical systems and can overcome the excessive dependence of model-based fault diagnosis methods on models. The artificial neural network has unique advantages for fault pattern recognition. Applying the theory and methods of artificial intelligence to mechanical fault diagnosis and developing intelligent mechanical fault diagnosis technology is a new way of performing mechanical fault diagnosis. The expert system for intelligent mechanical fault diagnosis has been widely used and has become an important direction of mechanical fault diagnosis. The research and development of hybrid intelligent design, control, monitoring, and diagnosis systems based on expert systems, neural networks, and fuzzy logic will become a major research hotspot. Although intelligent technology has been applied to all aspects of mechanical fault diagnosis, how to further promote and apply the existing advanced fault diagnosis equipment and technology and how to realize a low-cost, high-precision, and high-efficiency diagnosis system has become an urgent problem to be solved.

One problem with traditional methods is finding suitable signal features. These features can represent the characteristics of the signal, such as RMS, skewness kurtosis, etc. The features of different cases are also different and cannot be universal. The selection of several features is a difficult problem; too many or too few features will reduce the accuracy of diagnosis. Another problem is that if the fault categories are very close, diagnoses will be misclassified, resulting in lower accuracy. This research contribution proposes a method combining VMD and ResNets 101 for motor fault prediction and health management. The VMD method effectively simplifies the feature extraction process of motor fault diagnosis. All the subtle features of the signal can be presented on the frequency spectrum. Thus, even if the fault category is very close to the frequency spectrum, there are still different features. The data source of this research was verified by the motor database published by the Federal University of Rio de Janeiro (UFRJ). Six types of normal and faulty motor data were obtained from the database, and some types of faults were very similar. We converted all original motor databases into VMD time-frequency diagrams. Thus, VMD could display the time-frequency characteristics of different motor faults and find important features for fault diagnosis. Then, we used ResNets 101 to classify the image. The experimental results show that the accuracy of the VMD–ResNets 101 intelligent diagnosis method was 94% in the six categories. In the research, ResNets 101 was also compared with popular deep learning methods to confirm its high-accuracy recognition rate. In the results, VMD–ResNets 101 scored 19%, which was 8.3% higher than VMD–GoogLeNet and VMD–AlexNet. In the future, after building a model by analyzing data, our approach will be able to automatically learn features from the input data to predict and maintain faults in diagnostic equipment. This research has significant value for the maintenance and development of motors.

## 2. Research Methodology

VMD is an adaptive, completely non-recursive mode variation and signal processing method. This technology has the advantage of being able to determine the number of mode decompositions. Its adaptability lies in determining the number of mode decompositions of a given sequence according to the actual situation, and it can adaptively match the number of mode decompositions in the subsequent search and solution process. The approach has an optimal center frequency and limited bandwidth and can achieve the effective separation of intrinsic mode components (IMF) and perform signal frequency domain division. Then, the effective decomposition components of a given signal and the optimal solution of the variational problem were obtained.

The overall framework of variational mode decomposition was the variational problem, which mainly includes the structural variational problem and its solution. Regarding the motor data sequence as a non-stationary signal *f*, the variational problem was described as seeking K mode functions $u_{k}\left(t\right)\left(k=1,2,\cdots ,K\right)$. The *t* in 𝑢(𝑡) represents a function of time. Each mode has a finite bandwidth of the center frequency, meaning that the sum of the estimated bandwidth of each mode was the smallest. The constraint condition was that the sum of each mode was equal to the input signal *f*. The specific construction steps were as follows.

In the paper that introduces VMD [34], it is first defined as follows. The 1D Hilbert transform is the linear, shift-invariant operator H that maps all 1D cosine functions into their corresponding sine functions. It is an all-pass filter that is characterized by the transfer function:

$$\hat{h}\left(\omega \right)=-jsign\left(\omega \right)=-j\omega /\left|\omega \right|.$$

Thus, the Hilbert transform is a multiplier operator in the spectral domain. The corresponding impulse response is h(ω)=1/(πt).

For each mode function $u_{k}\left(t\right)$, its analytic signal is calculated through the Hilbert transform to obtain its one-sided spectrum:

$$\left(\delta \left(t\right)+\frac{j}{\pi t}\right)\cdot u_{k}\left(t\right)$$

where

(1) $$\delta \left(t\right)=\{\begin{array}{c} 0\;t\neq 0\\ \infty \;t=0 \end{array},\int_{-\infty }^{+\infty }\delta \left(t\right)dt=1$$

For each mode function $u_{k}\left(t\right)$, the frequency spectrum of each mode is modulated to the corresponding base band by aliasing the exponential term of its corresponding center frequency $\omega_{k}$:

(2) $$\left[\left(\delta \left(t\right)+\frac{j}{\pi t}\right)\cdot u_{k}\left(t\right)\right]e^{-j\omega_{k}t}$$

where $e^{-j\omega_{k}t}$ is the phasor description of the center frequency in the complex plane.

The bandwidth of $u_{k}\left(t\right)$ can be estimated by the Gaussian smoothing method of the above modulation signal; that is, the square $L^{2}$ norm of its gradient is calculated, and the solution can be expressed as a variational problem with constraints:

(3) $$\{\begin{array}{c} \underset{\left\{u_{k}\right\},\left\{\omega_{k}\right\}}{\min}\left\{\sum_{k=1}^{K}||\partial_{t}\left[\left(\delta \left(t\right)+\frac{j}{\pi t}\right)\cdot u_{k}\left(t\right)\right]e^{-j\omega_{k}t}||{}_{2}^{2}\right\}\\ s.t.\;\sum_{k=1}^{K}u_{k}=f \end{array}\;$$

where $\left\{u_{k}\right\}$ *=* $\left\{u_{1},\cdots ,u_{K}\right\},\left\{\omega_{k}\right\}$ *=* $\left\{\omega_{1},\cdots ,\omega_{K}\right\}$.

The solution to this variational problem is as follows: (1) The augmented Lagrangian function is introduced to transform the constrained variational problem into a non-constrained variational problem.

(4) $$L\left(\left\{u_{k}\right\},\left\{\omega_{k}\right\},\lambda \right)=\alpha \sum_{k=1}^{K}||\partial_{t}\left[\left(\delta \left(t\right)+\frac{j}{\pi t}\right)\cdot u_{k}\left(t\right)\right]e^{-j\omega_{k}t}||{}_{2}^{2}+||f\left(t\right)-\sum_{k=1}^{K}u_{k}{\left(t\right)||}_{2}^{2}+\lambda \left(t\right),f\left(t\right)-\sum_{k=1}^{K}u_{k}\left(t\right)\;\;\;$$

where α is the secondary penalty factor, and λ(t) is the Lagrangian multiplication operator.

(2) The optimal solution of Equation (4) is obtained by alternately updating $u_{k}^{n+1},\omega_{k}^{n+1}$, and $\lambda_{k}^{n+1}$ (n represents the number of iterations), where $u_{k}^{n+1}$ is obtained by Equation (5).

(5) $$u_{k}^{n+1}=arg\underset{u_{k}\in X}{\min}\left\{\alpha ||\partial_{t}\left[\left(\delta \left(t\right)+\frac{j}{\pi t}\right)\cdot u_{k}\left(t\right)\right]e^{-j\omega_{k}t}||{}_{2}^{2}+||f\left(t\right)-\sum_{k=1}^{Kk}u_{k}\left(t\right)+{\frac{\lambda \left(t\right)}{2}||}_{2}^{2}\right\}$$

where X is all the desirable sets of $u_{k}$.

(3) A Fourier equidistant transform is used to transform Equation (5) into the frequency domain, and the solution of the secondary optimization problem is obtained with Equation (6).

(6) $${\hat{u}}_{k}^{n+1}\left(\omega \right)=\frac{\hat{f}\left(\omega \right)-\sum_{i\neq k}\hat{u}\left(\omega \right)+\frac{\hat{\lambda }\left(\omega \right)}{2}}{1+2\alpha {\left(\omega -\omega_{k}\right)}^{2}}$$

(4) The minimum value of $\omega_{k}^{n+1}$ is determined in the same way, and the center frequency update problem is transformed to the frequency domain:

(7) $$\omega_{k}^{n+1}=arg\underset{\omega_{k}}{\min}\left\{\int_{0}^{\infty }{\left(\omega -\omega_{k}\right)}^{2}{\left|{\hat{u}}_{k}\left(\omega \right)\right|}^{2}d\omega \right\}$$

The calculation result of the center frequency is solved as shown in Equation (8):

(8) $$\omega_{k}^{n+1}=\frac{\int_{0}^{\infty }\omega {\left|{\hat{u}}_{k}\left(\omega \right)\right|}^{2}d\omega }{\int_{0}^{\infty }{\left|{\hat{u}}_{k}\left(\omega \right)\right|}^{2}d\omega }$$

where ${\hat{u}}_{k}^{n+1}\left(\omega \right)$ represents the current remaining amount $\hat{f}\left(\omega \right)-\underset{i\neq k}{\sum }{\hat{u}}_{i}\left(\omega \right)$ of Wiener filtering.$\;\omega_{k}^{n+1}$ represents the center of gravity of the power spectrum of the current mode function. An inverse Fourier transform on $\left\{{\hat{u}}_{k}\left(\omega \right)\right\}$ is performed, and the real part is $\left\{u_{k}\left(t\right)\right\}$.

ResNet was proposed in 2015 and won first place in the classification task of the ImageNet competition. Because it is a “simple and practical” method, many methods afterward have been based on ResNet50 or ResNet101, and these are widely used in the detection, segmentation, recognition, and other fields. It uses a connection method called “shortcut connection.” The bottom part of the ResNet model is the main part of the convolution. For the feature extraction of the input image, the convolution calculation must be performed to perform subsequent classification and mask and frame calculations. As the number of layers increased, convolutional networks enhanced feature expression capabilities, but they also faced the problem of vanishing gradients because the deeper the number of layers of the ordinary neural network, the closer the initialization parameter was to 0. Since neural network training usually uses the backpropagation algorithm for chain product derivation, when the shallow parameters are updated, as the information propagates forward, the gradient of the shallow layer tends to zero. Eventually, the gradient disappeared, causing the number of network layers to increase to a certain limit, and the accuracy of the model did not increase but decreased. The residual convolutional network (ResNet) [14,15,16] effectively avoided the problem of gradient disappearance by introducing a residual module and realized the improvement of model accuracy. Equation (9) is the mathematical expression of the residual module: the input data x_i of the residual blocks are mapped to the identity $W_{i}x_{i}$ through a shortcut connection (W=1 if no dimension conversion is required). At the same time, $x_{i}$ is convolved and activated by a linear correction unit (ReLU) $G_{i}$, and the residual value $F(x_{i},G_{i})$ is output:

(9) $$x_{i+1}=F(x_{i},G_{i})+W_{i}x_{i}$$

where $x_{i}$ is the data input of the ith layer residual block, $G_{i}$ is the activation function, $F(x_{i},G_{i})$ is the residual value, W is the identity mapping parameter of the shortcut link (usually a constant 1), and $x_{i+1}$ is the input of the i+1 layer residual block.

Any $x_{i}$ deeper than $x_{k}$ can be represented by $x_{i}$ (see Equation (10)). The gradient of the loss function to $x_{i}$ is dl/dx, which can be expressed as shown in Equation (11), and W takes a value of 1:

(10) $$x_{k}=\sum_{j=i}^{k-1}F(x_{i},G_{i})+x_{i}\;\;$$

(11) $$\frac{dl}{dx_{i}}=\frac{dl}{dx_{k}}\frac{dx_{k}}{dx_{i}}$$

where $x_{k}$ is the input value of the residual block of the k-th layer, k>i. $x_{j}$ is the residual value from layer *i* to layer k−1. $G_{j}$ is the activation function from layer *i* to layer k−1. $F(x_{j},G_{j})$ is the residual value from level *i* to level k−1. Equation (10) is substituted into Equation (11) to obtain Equation (12). It can be seen from Equation (12) that the gradient of the deeper layer $x_{k}$ can be transferred to any shallower layer $x_{i}$. Additionally, the product calculation in the conduction process is addition, thus no matter how deep the network layer is, its gradient will never disappear.

(12) $$\begin{array}{ll} \frac{dl}{dx_{i}} & =\frac{dl}{dx_{k}}\frac{d(\sum_{j=i}^{k-1}F(x_{j},G_{j})+x_{i})}{dx_{i}}\\ & =\frac{dl}{dx_{k}}(1+\frac{d(\sum_{j=i}^{k-1}F(x_{j},G_{j})}{dx_{i}} \end{array}$$

## 3. Database Description

The data obtained in this study provided test data for normal and faulty motors, all of which were taken from the website of the Federal University of Rio de Janeiro at http://www02.smt.ufrj.br/~offshore/mfs/. Html (accessed on 10 September 2021). These time series were acquired by SpectraQuest’s Mechanical Failure Simulator (MFS) aligned with the sensors on the balanced vibration (ABVT). The series contains six different kinds of test data: normal function, unbalanced fault, horizontal and vertical misalignment fault, and internal and external bearing malfunction. Table 1 shows the specifications of the experimental equipment. Table 2 shows the motor fault conditions and the number of experiments.

The data acquisition system was an industrial IMI sensor—a Model 601A01 accelerometer in radial, axial and tangential directions—and the specifications were as follows:

o Sensibility: (±20%) 100 mV/g (10.2 mV/(m/s^2^));
o Frequency range: (±3 dB) 16–600,000 CPM (0.27–10,000 Hz);
o Measurement range: ±50 g (±490 m/s^2^).

A National Instruments NI 9234 device with four-channel analog acquisition modules was used with a sample rate of 51.2 kHz.

1. Normal sequence

There were 48 sequences without any failure, and the speed of each sequence was fixed, ranging from 737 rpm to 3686 rpm, with a step length of about 60 rpm.

2. Unbalanced fault

A load value of 10 g was used for the test, assuming the rotation frequency was within the same 48 values used under normal operating conditions.

3. Horizontal parallel misalignment

By moving the motor shaft horizontally by 2.0 mm, this type of fault was placed on the test platform. For each horizontal displacement, we used the same rotation frequency range as in normal operation.

4. Vertical and parallel misalignment

By moving the motor shaft 1.90 mm horizontally, this type of fault was placed on the test platform. For each vertical shift, the same rotation frequency range as in normal operation was used.

5. Thirty-five-gram failure of bearing outer track

As one of the most complex components of the machine, rolling bearings are the most prone to failure components. Bearings may have defective components (outer track, rolling elements, and inner track). When there is no imbalance, bearing failure is imperceptible. Therefore, the 35 g mass of the faulty outer track was designed to induce a detectable effect with a different rotation frequency than before.

6. Bearing cage fault: 6 g quality failure

In this design, another fault—overhang bearing—involved a cage fault of 6 g of mass, which was used to induce a detectable effect.

## 4. Results and Discussion

**Figure 1:**
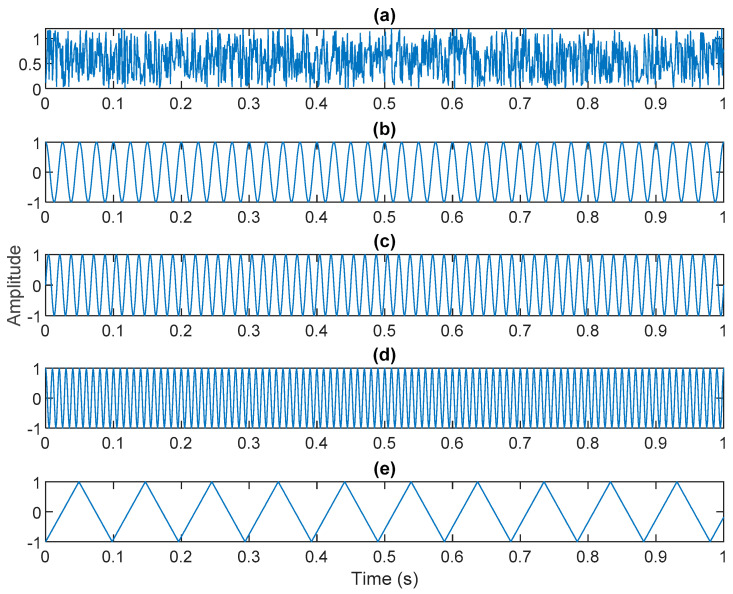
Original sources: (**a**) white Gaussian noise; (**b**) 40 Hz cosine waveform; (**c**) 60 Hz sine waveform; (**d**) 100 Hz sin waveform; (**e**) 10 Hz triangular waveform.

**Figure 2:**
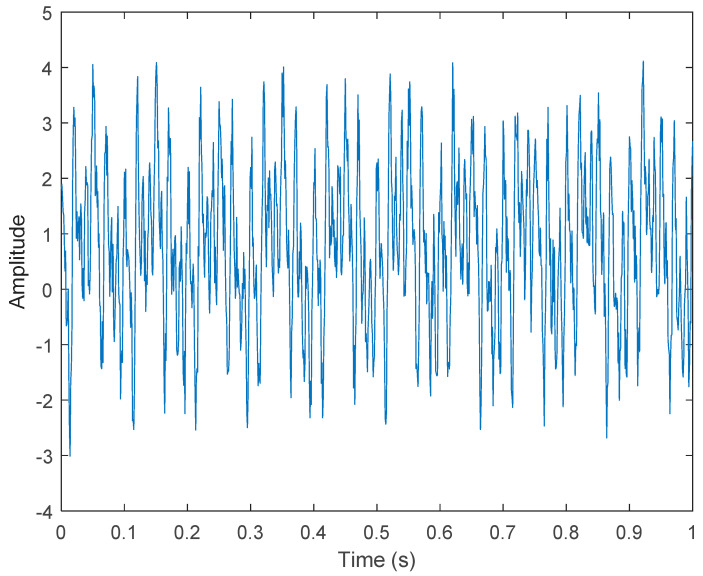
Simulated motor fault data, including four-fault characteristics and Gaussian noise.

The periodic impact energy caused by the early failure of the electric vehicle motor is weak, and it is relatively difficult to extract the fault characteristics due to the influence of environmental noise and data attenuation. In this article, we attempted to use the VMD method to analyze the early failure data of motor bearings. We first verified the performance and effectiveness of VMD. There were five different characteristics of data $s_{1}\left(t\right)$, $s_{2}\left(t\right)$, $s_{3}\left(t\right)$, $s_{4}\left(t\right)$, and $s_{5}\left(t\right)$ in the simulation. $s_{1}\left(t\right)$ represents Gaussian white noise. In addition, the fault data $s_{2}\left(t\right)$, $s_{3}\left(t\right)$, $s_{4}\left(t\right)$, and $s_{5}\left(t\right)$ of the electric vehicle motor were 40, 60, and 100 Hz sine and cosine waveforms and 10 Hz triangular waveforms, respectively, as shown in Figure 1. After mixing, four of the original sources were contaminated by Gaussian white noise, as shown below:

(13) $$x\left(t\right)=1.56*s_{1}\left(t\right)+s_{2}\left(t\right)+s_{3}\left(t\right)+s_{4}\left(t\right)+s_{5}\left(t\right)$$

where $s_{1}\left(t\right)$ represents Gaussian white noise. To increase the noise, we increased the gain value by 1.56. $s_{2}\left(t\right)=cos\left(2\times \pi \times 40\times t\right),s_{3}\left(t\right)=sin\left(2\times \pi \times 60\times t\right)$, $s_{3}\left(t\right)=cos\left(2\times \pi \times 100\times t\right)$. $s_{5}\left(t\right)$ represents a triangular wave of 10 Hz. x(t) is shown in Figure 2.

**Figure 3:**
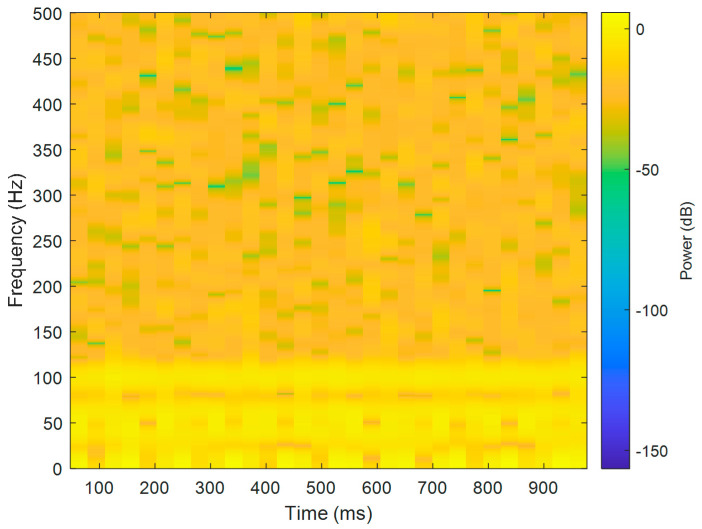
The results of Fourier time-frequency analysis of simulated motor fault data.

The short-term Fourier transform (STFT) is a time-frequency analysis method with a fixed time window. The basic idea was to use a window function to intercept the signal. If a signal was stable in the window, a Fourier transform was used to analyze the signal in the window. The frequency that existed at a time was determined, and then the window function was moved along the signal time to obtain the relationship between the signal frequency and time; the time–frequency distribution is shown in Figure 3. The X-axis is time in seconds, the Y-axis is frequency, and the Z-axis color represents power (dB). Figure 3 shows that most of the data were disturbed by noise, and the 40, 60, and 100 Hz signals can be vaguely seen.

The following describes the VMD algorithm flow:

(1) Initialize $\left\{{\hat{u}}_{k}^{1}\right\},\left\{\omega_{k}^{1}\right\}$ and $\lambda^{1}$ and set the number of iterations to 1;

(2) Update ${\hat{u}}_{k}$ and $\omega_{k}$ according to Equation (6) and Equation (8);

(3) Update λ according to Equation (14);

(14) $$\lambda^{n+1}\left(\omega_{k}\right)\leftarrow \lambda^{n}\left(\omega_{k}\right)+\tau \left[f\left(\omega_{k}\right)-\sum_{k}{\hat{u}}_{k}^{n+1}\left(\omega_{k}\right)\right]$$

where τ is the noise tolerance parameter. When the signal contains strong noise, in order to achieve a good denoising effect, τ can be set to 0, but in practical applications, considering the distortion caused by denoising, we chose a value of τ=0.3 on the basis of many experiments.

(4) Given the discrimination accuracy ε>0, judge whether the convergence condition of Equation (15) is satisfied, and if it is satisfied, stop the iteration; otherwise, n increases to n+1 and return to step 2.

(15) $$\sum_{k=1}^{k}||{\hat{u}}_{k}^{n+1}-{\hat{u}}_{k}^{n}||{}_{2}^{2}/||{\hat{u}}_{k}^{n}||{}_{2}^{2}<\varepsilon$$

Compared with the original data of the motor, the data decomposed by VMD had stronger regularity, which can improve the accuracy of prediction.

**Figure 4:**
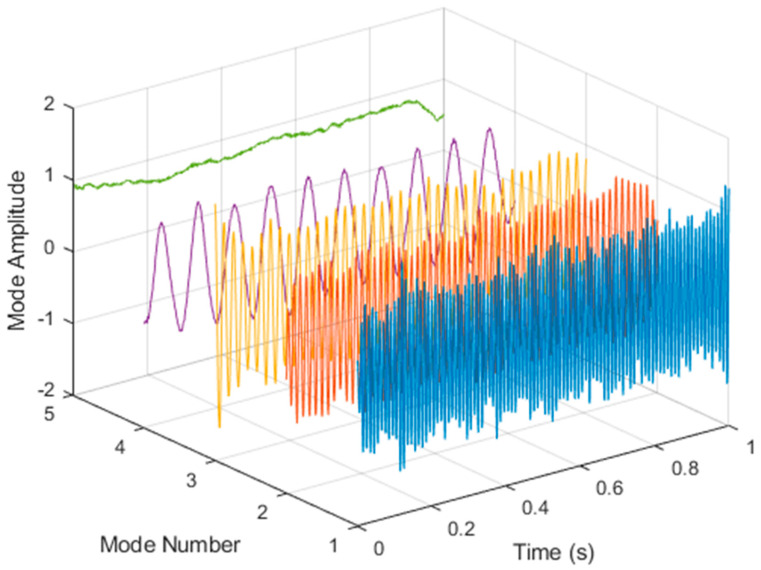
Five components of the VMD analysis result of simulated motor fault data.

**Figure 5:**
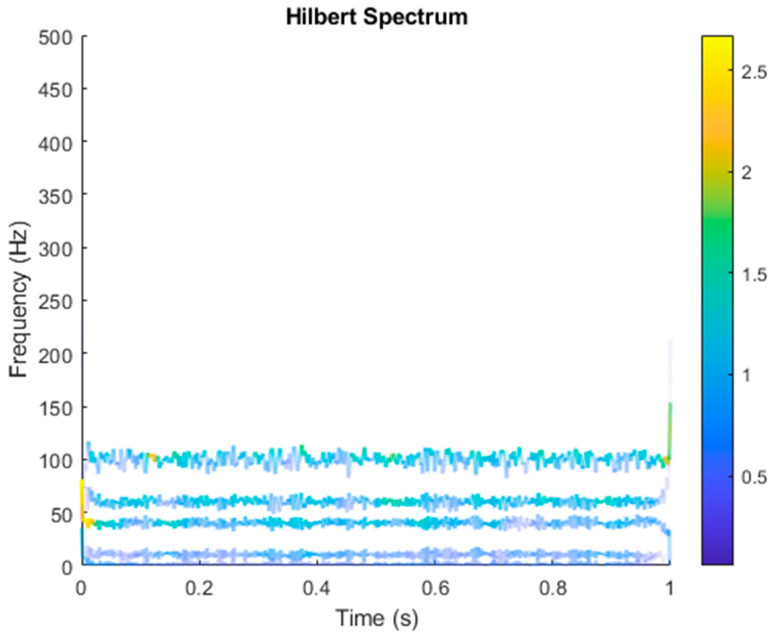
Hilbert conversion of five IMFs of simulated motor fault data after VMD analysis results.

After VMD was used to analyze x(t), the IMF components obtained were then subjected to a Hilbert transform, and the Hilbert marginal spectrum was obtained. Using VMD to decompose *x*(*t*), five mode components could be obtained, as shown in Figure 4. They were denoted as $u_{1}$,$\;u_{2}$, $u_{3}$, $u_{3}$, $u_{4}$, and $u_{5}$ and were very close to the components of the original signal. These five components were transferred to the Hilbert spectrum, as shown in Figure 5. This figure clearly shows the 10 Hz triangle wave and 40, 60, and 100 Hz signals at the bottom of the four components. VMD clearly appeared in the recombined signal, and there was no omission of frequency information. In addition, in the spectrogram of the recombined signal, the high-frequency signal generated by the noise was much flatter than the original signal. This shows that VMD can effectively retain useful information and remove noise.

**Figure 6:**
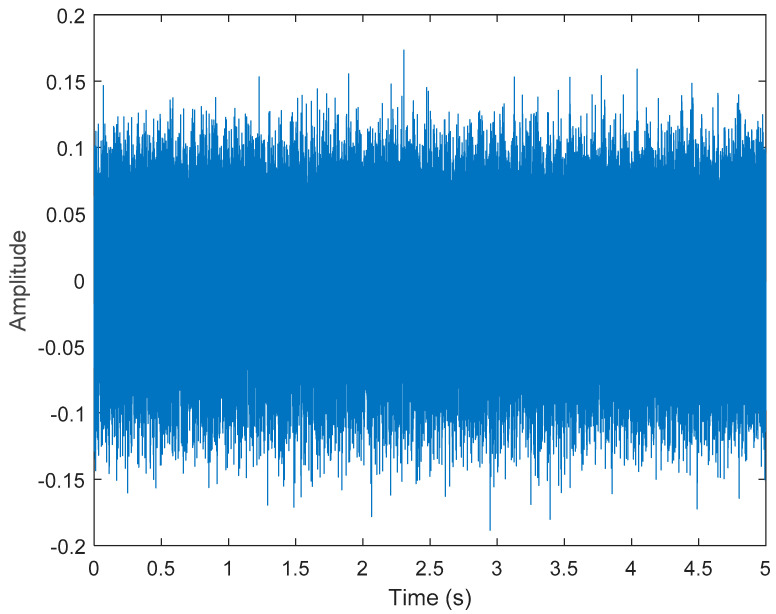
Vibration data of a normal motor.

**Figure 7:**
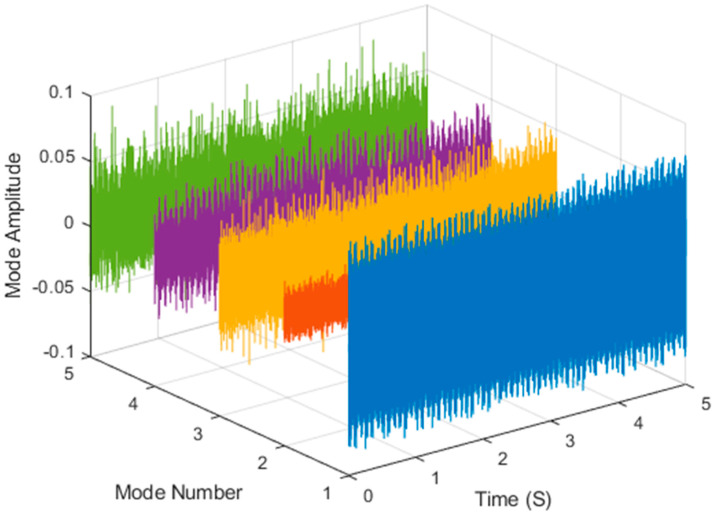
VMD analysis of the vibration normal motor data.

**Figure 8:**
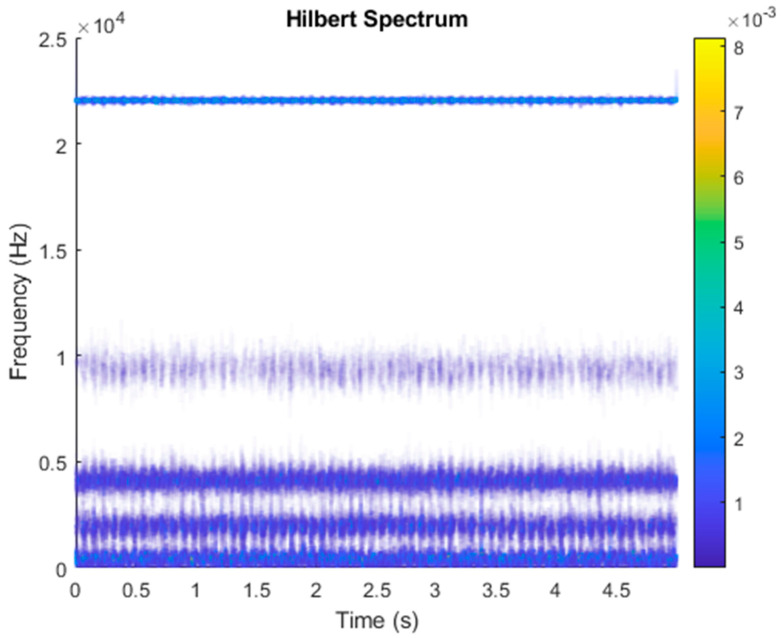
Hilbert transformation of VMD analysis of vibration data of a normal motor.

It is explained here that VDM was used to analyze real data. Normal (no fault) data had 48, a horizontal misalignment of 2.0 mm had 49, a vertical misalignment of 1.90 mm had 50, an imbalance of 10 g had 48, the underhang of the bearing in the outer race of 35 g had 37, and for the overhang bearing, which was a cage fault of 6 g, there were 49. In this study, all six types of data were analyzed by VMD and converted into the Hilbert spectrum. In the VMD parameter setting description, Max Iterations refers to the maximum number of optimization iterations, which was 500 times. Max Iterations was one of the stopping criteria for optimization; that is, when the number of iterations was greater than this number, the optimization was stopped. NumIMF (the number of extracted IMFs) was five IMFs. InitialIMFs (initial IMF) was a zero matrix, and PenaltyFactor (penalty factor) was 1000. This parameter was used to determine the fidelity of the reconstruction. Using a smaller penalty factor value can enable tighter data fidelity. LMUpdateRate (the update rate of the Lagrangian multiplier) was 0.01, which was the update rate of the Lagrangian multiplier in each iteration. A higher rate would lead to faster convergence, but it would increase the optimization process into a local best opportunity method. The InitializeMethod (the method of initializing the center frequency) was peaks, and “peaks” were used to initialize the center frequency to the peak position of the signal in the frequency domain. Figure 6 shows the vibration data of a normal motor. First, VMD decomposition of the normal motor data was performed, as shown in Figure 7. The result is shown in Figure 8 for the Hilbert marginal spectrum of each IMF. It can be clearly seen from Figure 8 that the IMF Hilbert marginal spectrum of the vibration data processed by VMD had a higher frequency resolution. There were five frequencies for normal motors, the most obvious of which were 22k Hz, 9452 Hz, 4512 Hz, 2077 Hz, and 550 Hz.

**Figure 9:**
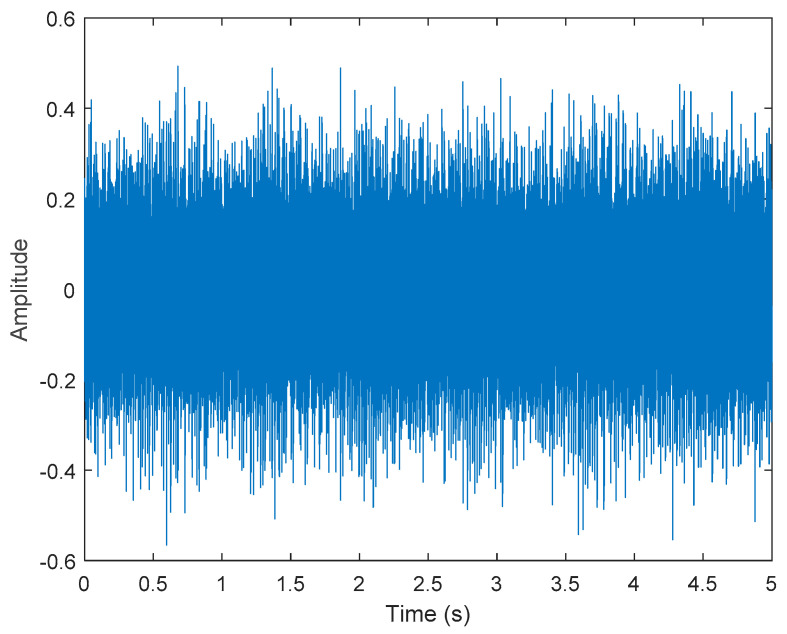
Vibration data of horizontal misalignment motor failure.

**Figure 10:**
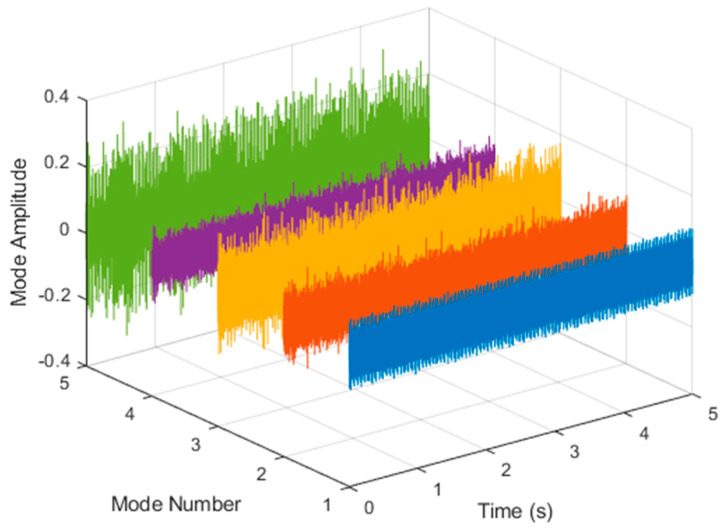
VMD analysis of vibration data of horizontal misalignment motor failure.

**Figure 11:**
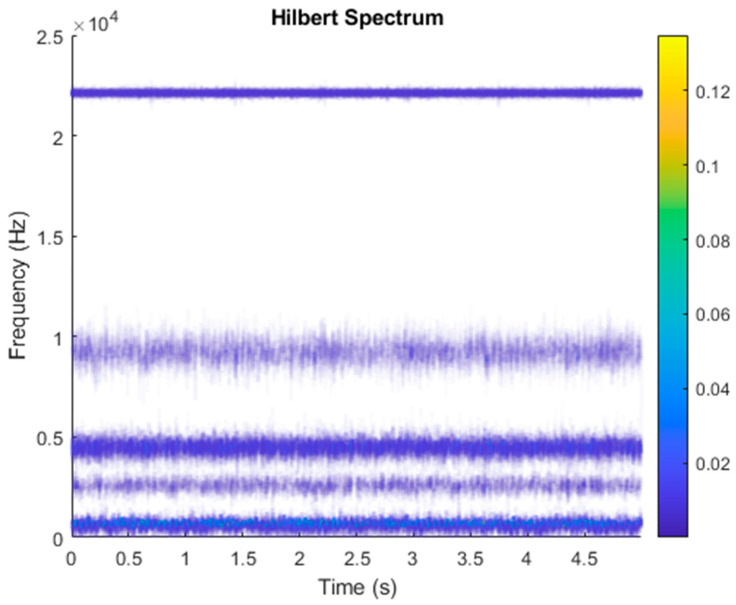
Hilbert transformation of VMD analysis of vibration data of horizontal misalignment motor failure.

Figure 9 shows the vibration data of the horizontal misalignment motor failure. First, we performed the VMD decomposition of horizontal misalignment motor fault data, as shown in Figure 10. The Hilbert transform was performed on each IMF component obtained after VMD processing, and the result of obtaining the Hilbert marginal spectrum is shown in Figure 11. It can be clearly seen from Figure 11 that the IMF Hilbert marginal spectrum of the vibration data processed by VMD had a higher frequency resolution. The horizontal misalignment motor fault had five frequencies, the most obvious of which were 22k Hz, 9300 Hz, 4400 Hz, 2600 Hz, and 657 Hz.

**Figure 12:**
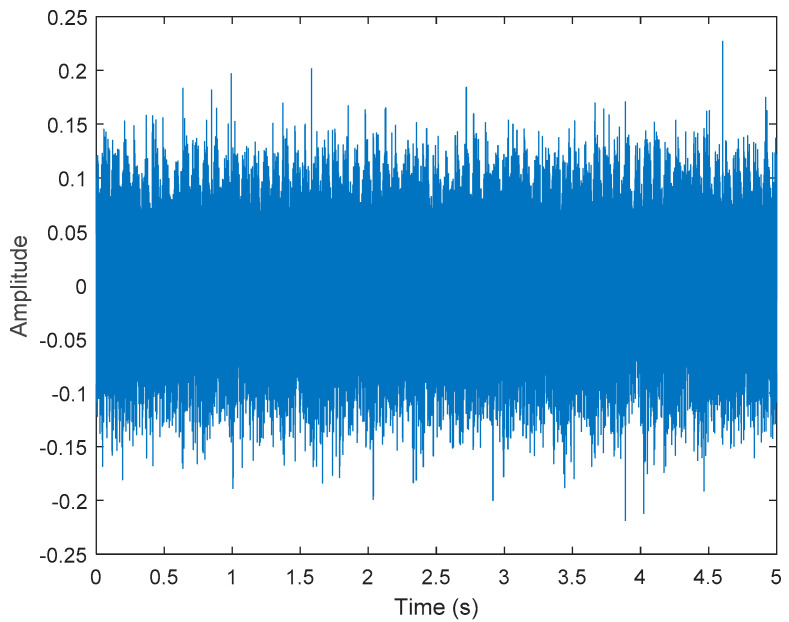
Vibration data of imbalance motor failure.

**Figure 13:**
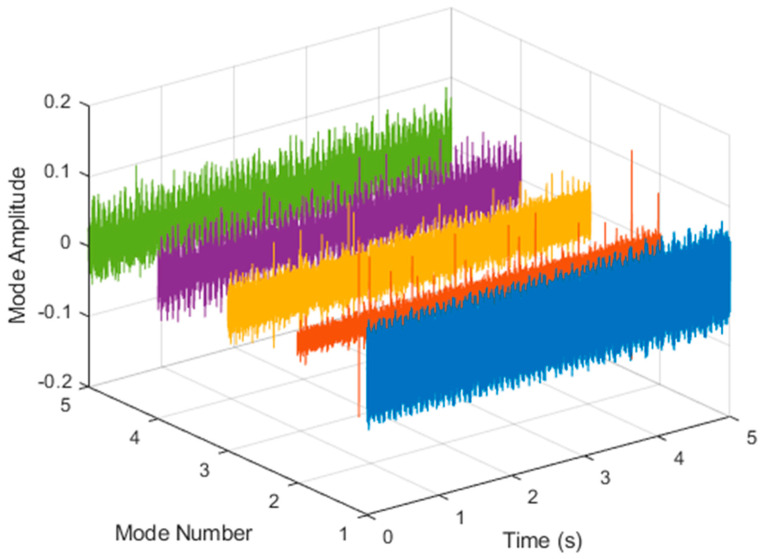
VMD analysis of vibration data of imbalance motor failure.

**Figure 14:**
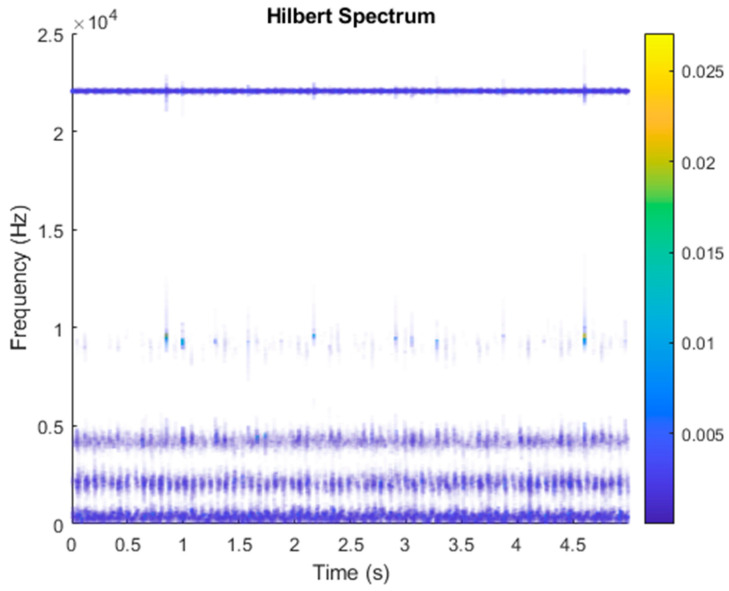
Hilbert transformation of VMD analysis of vibration data of imbalance motor failure.

Figure 12 shows the vibration data of the imbalance motor failure. First, we performed VMD decomposition for the imbalance motor fault data, as shown in Figure 13. A Hilbert transform was performed on each IMF component obtained after VMD processing, and the result of obtaining the Hilbert marginal spectrum is shown in Figure 14. It can be clearly seen from Figure 14 that the IMF Hilbert marginal spectrum of the vibration data processed by VMD has a higher frequency resolution. There were five frequencies for imbalance motor failures, with the most obvious being 22k Hz, 9100 Hz, 4260 Hz, 2120 Hz, and 364 Hz.

**Figure 15:**
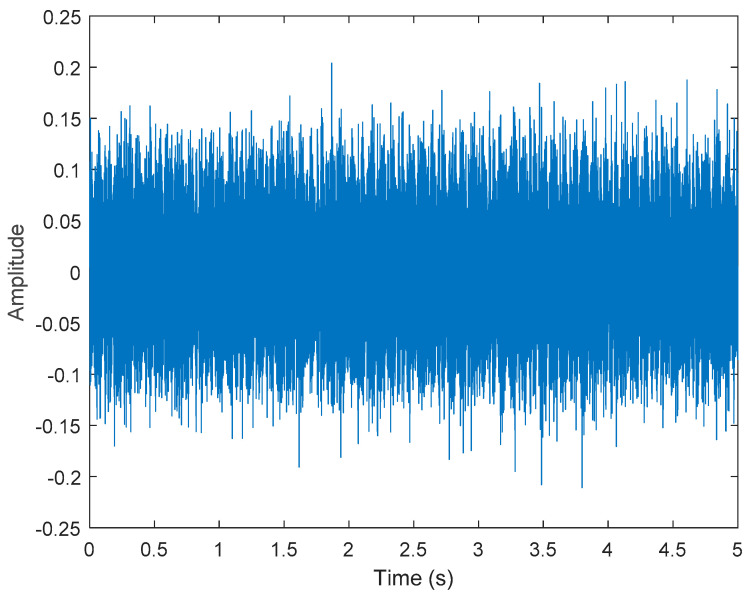
Vibration data of overhang bearing motor failure.

**Figure 16:**
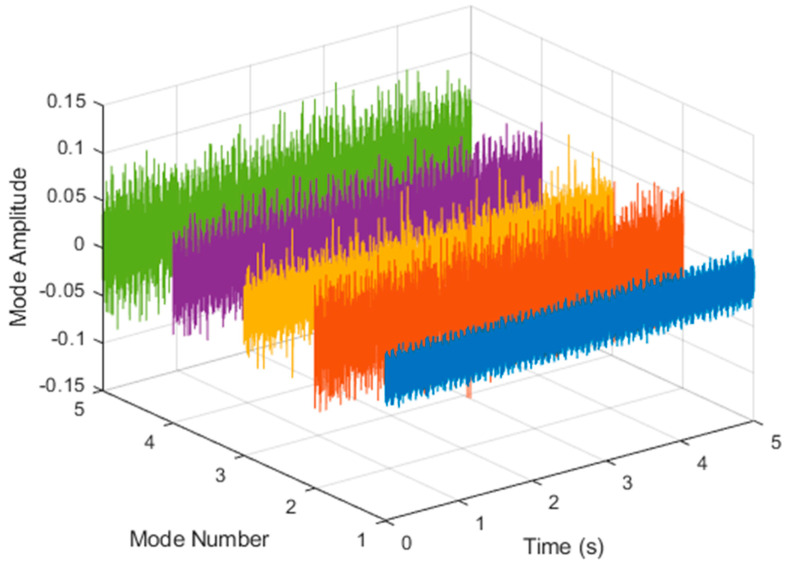
VMD analysis of vibration data of overhang bearing motor failure.

**Figure 17:**
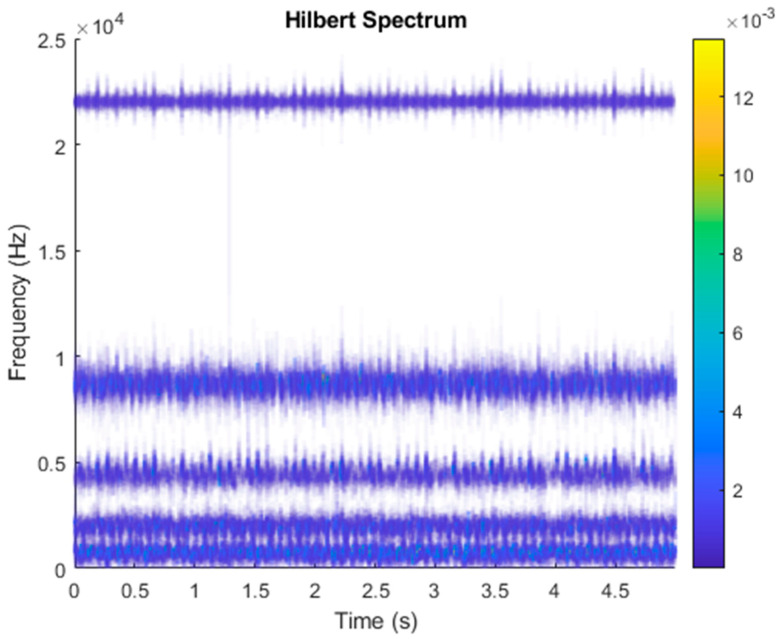
Hilbert transformation of VMD analysis of vibration data of overhang bearing motor failure.

Figure 15 shows the vibration data of an overhang bearing motor failure. First, the VMD decomposition of the overhang bearing motor fault data are shown in Figure 16. The Hilbert transform was performed on each IMF component obtained after VMD processing, and the Hilbert marginal spectrum was obtained. The result is shown in Figure 17. It can be clearly seen from Figure 17 that the IMF Hilbert marginal spectrum of the vibration data processed by VMD had a higher frequency resolution. There were five frequencies for overhang bearing motor failures, the most obvious of which were 22k Hz, 8800 Hz, 4500 Hz, 2000 Hz, and 656 Hz.

**Figure 18:**
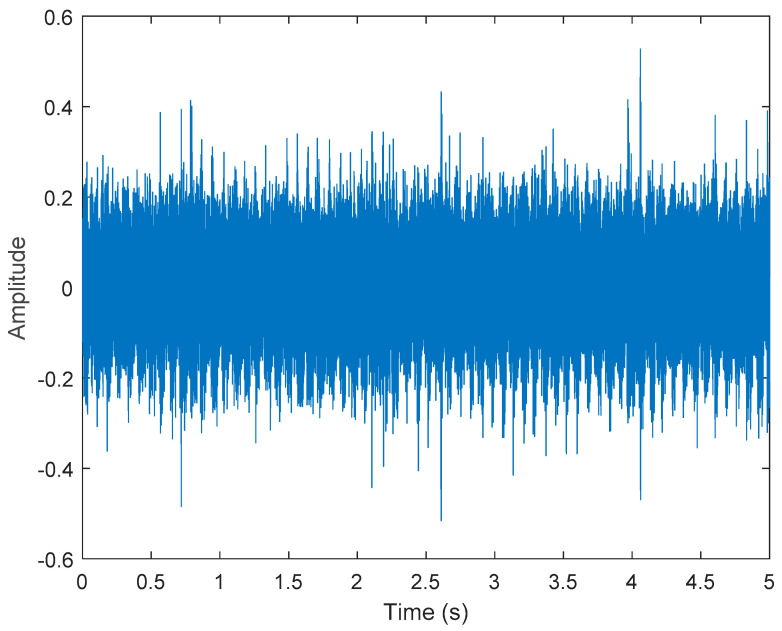
Vibration data of underhung bearing motor failure.

**Figure 19:**
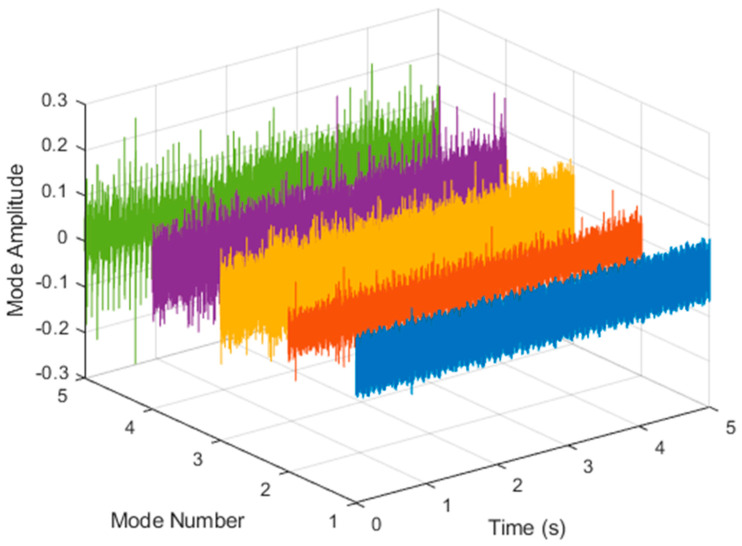
VMD analysis of vibration data of underhung bearing motor failure.

**Figure 20:**
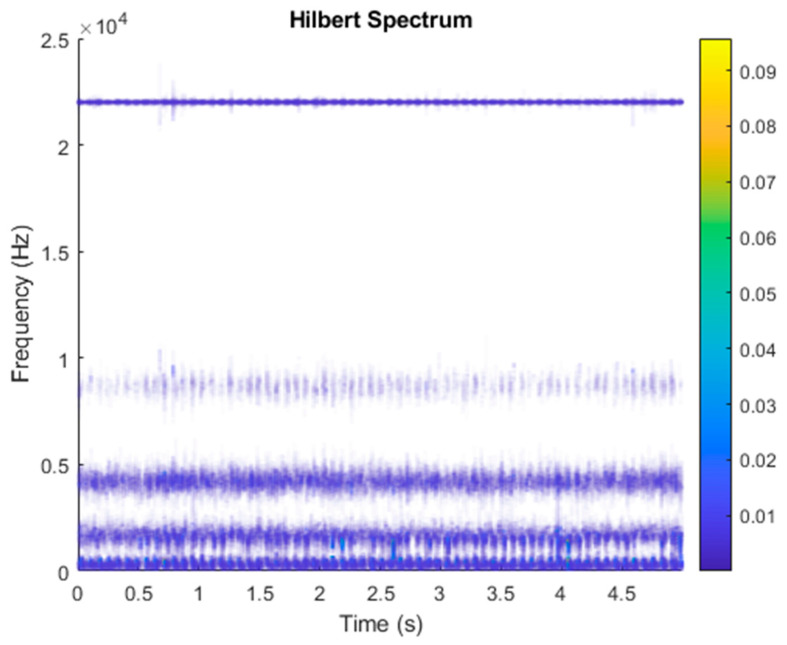
Hilbert transformation of VMD analysis of vibration data of underhung bearing motor failure.

Figure 18 shows the vibration data of an underhung bearing motor failure. First, the VMD decomposition of the underhung bearing motor fault data is shown in Figure 19. The Hilbert transform was performed on each IMF component obtained after VMD processing, and the Hilbert marginal spectrum was obtained. The result is shown in Figure 20. It can be clearly seen from Figure 20 that the IMF Hilbert marginal spectrum of the vibration data processed by VMD had a higher frequency resolution. There were five frequencies for underhung bearing motor failure, the most obvious of which were 22k Hz, 8800 Hz, 4500 Hz, 2000 Hz, and 656 Hz.

**Figure 21:**
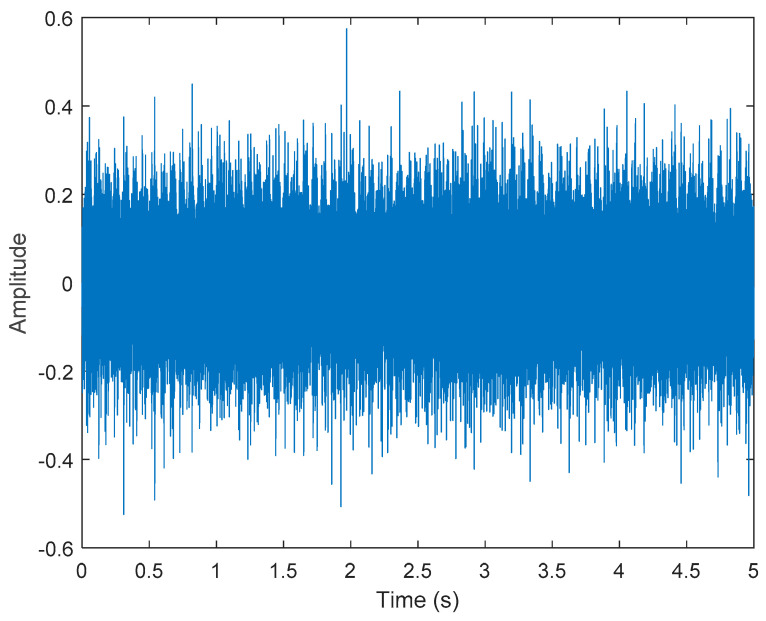
Vibration data of vertical misalignment motor failure.

**Figure 22:**
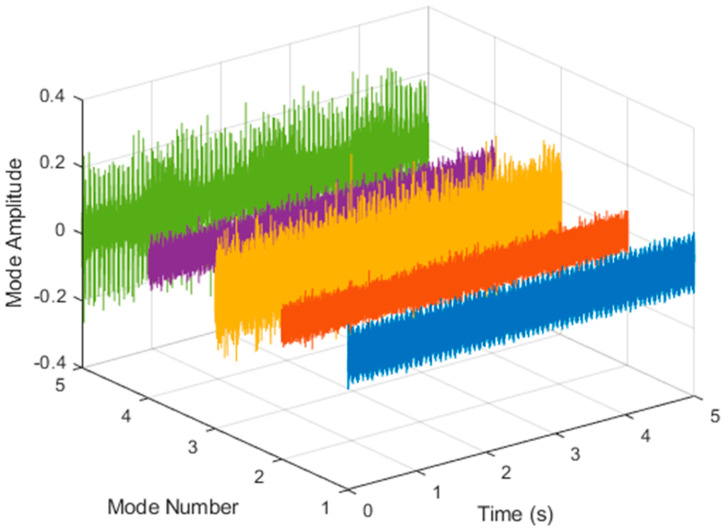
VMD analysis of vibration data of vertical misalignment motor failure.

**Figure 23:**
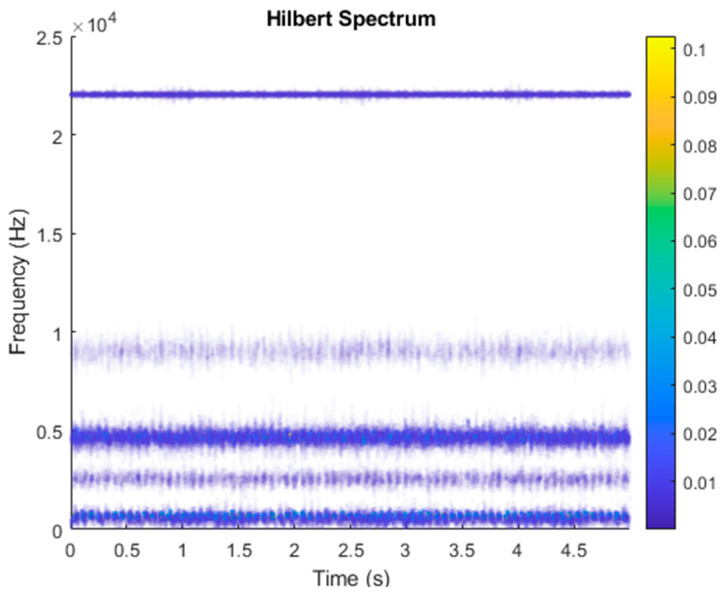
Hilbert transformation of VMD analysis of vibration data of vertical misalignment motor failure.

Figure 21 shows the vibration data of the vertical misalignment motor failure. First, we performed the VMD decomposition of the vertical misalignment motor fault data, as shown in Figure 22. The Hilbert transform was performed on each IMF component obtained after VMD processing, and the Hilbert marginal spectrum was obtained. The result is shown in Figure 23. It can be clearly seen from Figure 23 that the IMF Hilbert marginal spectrum of the vibration data processed by VMD had a higher frequency resolution. The vertical misalignment motor fault had five frequencies, the most obvious of which were 22k Hz, 8800 Hz, 4500 Hz, 2000 Hz, 656 Hz.

The above normal and five different types of motor faults were decomposed by VMD, and each IMF component was obtained by a Hilbert transform. Although the obtained Hilbert marginal spectra were different, the five frequencies were very close. Engineers without professional training cannot understand whether there is a fault or what kind of fault they are witnessing the first time they experience this. To evaluate the method proposed in the text more comprehensively, it was compared with the current mainstream methods with the same test set from both qualitative and quantitative aspects. In this study, all data in the six categories were converted into images of the Hilbert spectrum. Then, we used three deep learning image classification methods for identification. The three deep learning image classification methods were AlexNet, GoogLeNet, and ResNet-101, and we determined the method with the highest recognition rate. In total, 70% of all images were used for training, and 30% were used for verification. The number of images for training and verification is shown in Table 3.

The following explains the results in the confusion matrix. The rows correspond to the results of the predicted class, and the columns correspond to the results of the true class. The diagonal cells correspond to the correctly classified observations. The diagonal cells correspond to the predictions of the correct classification. Off-diagonal units correspond to predictions of misclassification. Each cell shows the number of forecasts and the percentage of the total number of forecasts. The column on the far right of the graph shows the percentage of correct and incorrect classifications of all examples whose predicted results belong to each category. These indicators are usually called the accuracy (or positive predictive value) and false discovery rate, respectively. The row at the bottom of the graph shows the percentage of correct and incorrect classifications of all examples belonging to each category. These indicators are usually called the recall rate (or true positive rate) and false negative rate, respectively. The cell in the lower right corner of the graph shows the overall accuracy.

In this research, we hoped to find a deep learning algorithm for classification with high accuracy because deep learning methods have different classification accuracies due to different use cases. In this research, we used many algorithms and finally selected these three popular algorithms to illustrate the comparison. In the discussion of the results, Table 3 is also added to show the network structure of the three methods, Table 4 shows the comparison of the parameters of the three methods, and Table 5 describes the comparison of the characteristics of the three methods with additional explanations.

In 2012, AlexNet [38], proposed by Alex Krizhevsky and others, won the ImageNet competition with a great advantage. The AlexNet feature has the advantage of using ReLU because it has a fast convergence speed. Compared with Sigmoid or Tanh, ReLU saves complex operations and only requires a threshold to obtain the activation value. Another advantage is the use of dropout and data augmentation to reduce overfitting.

GoogLeNet [39] was the winner of the ImageNet competition in 2014. There are three characteristics of this method:

1. The main feature of this network architecture is that it improves the utilization of the internal computing resources of the network;
2. It increases the depth and width of the network—the network depth reaches 22 layers (not including the pooling layer and the input layer), but without increasing the computational cost;
3. The approach uses the Network in Network method to increase the performance of the network. This method can be seen as an additional 1*1 convolutional layer plus a ReLU layer.

ResNet [37] was the champion of the ImageNet competition in 2015 and reduced the error rate of image classification recognition to 3.6%. This result even exceeded the accuracy of normal human eye recognition. The overall architecture of ResNet can be divided into three parts:

1. Input stem: general convolution is used along with a large stride to reduce the resolution;
2. Stage block: ResNet has four stage blocks, and each stage block is made up of several building blocks. Whether using stride or pooling, each stage generally reduces the resolution and enlarges the width (channel) first, and then performs a series of residual learning;
3. Output stem: according to the task, different outputs are designed. Generally speaking, this side will change with the task, thus it is usually not counted in the backbone of ResNet.

Since the resolution and network width of the connection between the input of the first building block and the residual path of each stage are different, the first block will have an additional convolution to adjust the resolution and width. The characteristics of these three deep learning methods are compared in Table 5.

A comparison of the number of networks, type, size, parameters (millions), and image input size is presented in Table 3. Regarding the number of networks, ResNet101 has 101 at most, and GoogLeNet has 22 at least. The type of the AlexNet approach is a series. A series network is a neural network for deep learning with layers arranged one after the other. GoogLeNet and ResNet101 are directed acyclic graph (DAG) networks for deep learning. A DAG network is a neural network for deep learning with layers arranged as a directed acyclic graph. A DAG network can have a more complex architecture in which layers have inputs from multiple layers and outputs to multiple layers.

To compare the three deep learning methods in a fair situation, the parameters listed in Table 4 were adopted. There are several major training options in the selection of solver algorithms. Mini-batch options included the maximum epochs, mini-batch size, and shuffle. The validation section included validation frequency and validation patience. The solver options included the initial learn rate, learn-rate schedule, mini-batch size, learn rate drop factor, L2 regularization, and momentum. The hardware option was a GPU.

**Figure 24:**
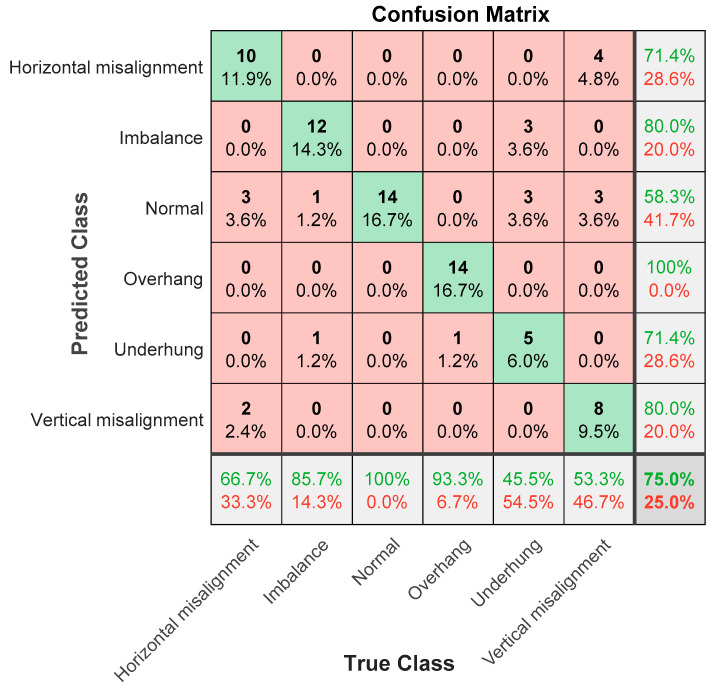
AlexNet classification results in confusion matrix; the accuracy rate is 75%.

Firstly, it is introduced that AlexNet generally uses non-linear functions such as Sigmoid or tanh as excitation functions in traditional neural networks. However, they are prone to gradient dispersion or gradient saturation. Taking the Sigmoid function as an example, when the input value is very large or very small, the gradient of these neurons is close to 0 (gradient saturation phenomenon). If the initial value of the input is large, the gradient needs to be multiplied by a sigmoid derivative when backpropagation. This will cause the gradient to become increasingly small, making the network difficult to learn. In AlexNet, the ReLU (rectified linear units) excitation function is used. The equation of this function is F(x)=max(0,x), when the input signal is less than 0, the output is 0. When the input signal is greater than 0, the output is equal to the input, and ReLU is used instead of Sigmoid/tanh. Since ReLU is linear and the derivative is always 1, the amount of calculation is greatly reduced, and the convergence speed will be much faster than Sigmoid/tanh. Figure 24 shows the AlexNet classification result, and the accuracy rate is 75%. In the six categories, the classification accuracy rate is 100% for overhang bearing, the lowest normal is 58.3%, and the error rate is 12.5% for horizontal misalignment, 4.16% for imbalance, 12.5% for underhang bearing, and 12.5% for vertical misalignment.

**Figure 25:**
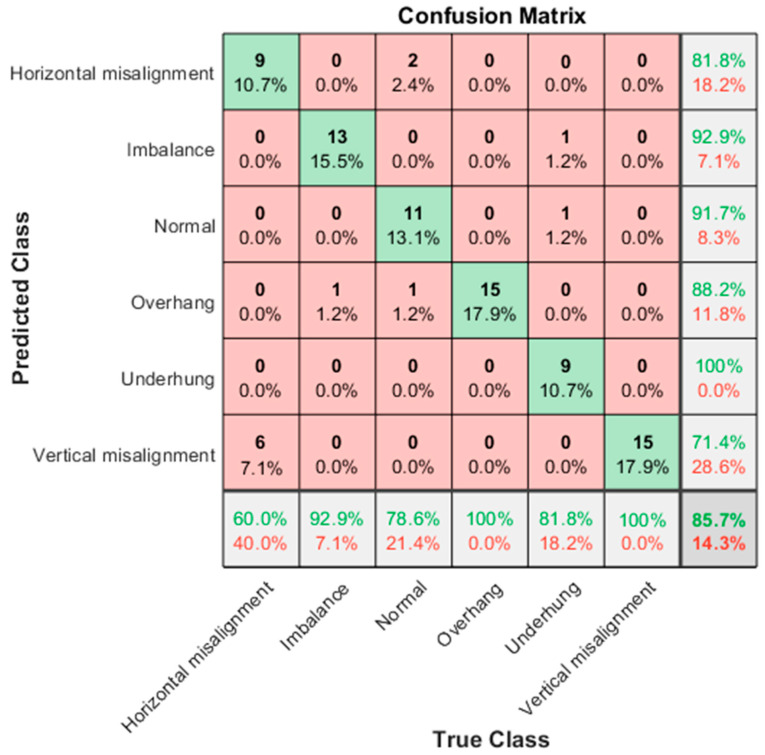
GoogLeNet classification results in confusion matrix; the accuracy rate is 85.7%.

The “Inception module” proposed in the GoogLeNet model adopts a “Split–Transform–Merge” strategy for network design, which can fuse information of different scales, enhance the expression ability of the model, and improve the performance of the model. Its number of training parameters is also several times less than AlexNet, and the accuracy is better. GoogLeNet stacks three types of convolutions with one pooling, which increases the width of the network. After being stacked in this way, more detailed information and features of the input image can be captured. GoogLeNet has the following different characteristics: 1. The pure convolutional layer and pooling layer are changed to the Inception architecture; 2. In the final classification, average pooling is used to replace the fully connected layer; 3. The network includes two auxiliary classifiers to avoid the disappearance of the gradient. Figure 25 shows the GoogLeNet classification result, and the accuracy rate is 85.7%. In the six categories, the classification accuracy rate is 100% for underhang bearing; the lowest is vertical misalignment, for which the classification accuracy rate is 71.4%, and the error rate for horizontal misalignment is 28.6%.

**Figure 26:**
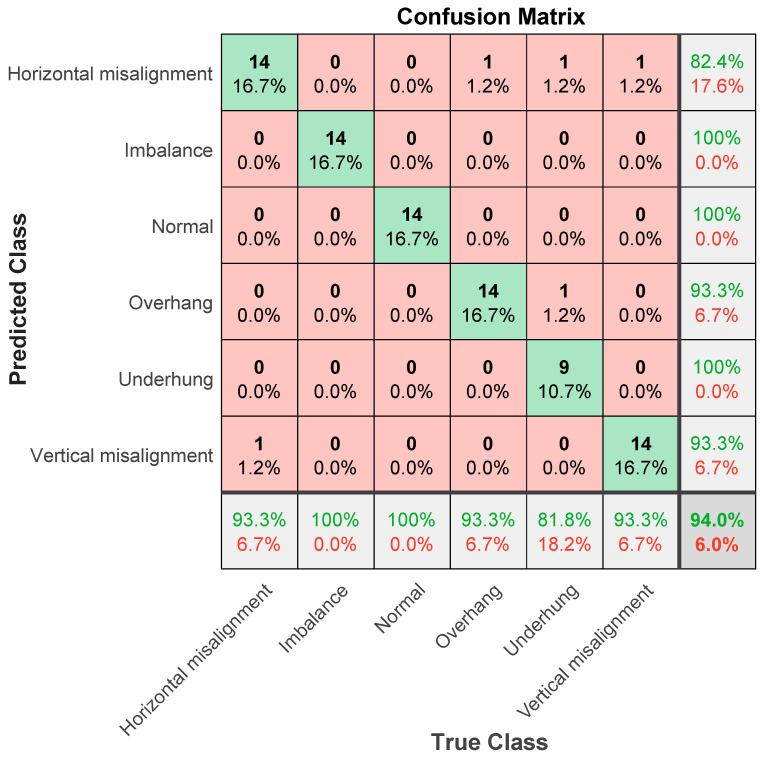
ResNet101 classification results in confusion matrix; the accuracy rate is 94%.

As the network deepens, the accuracy of the training set decreases. Therefore, researchers have proposed a brand-new network for this problem, called a deep residual network. In this context, a brand-new structure is introduced, such as the residual module in the ResNet-101 model. This effectively solves the gradient dispersion, gradient explosion, and degradation problems caused by the deepening of the neural network layer. In theory, for the problem of “decreasing accuracy as the network deepens,” ResNet provides two options: namely identity mapping and residual mapping. If the network has reached the optimum, the approach continues to deepen the network, and the residual mapping will be pushed to 0, leaving only the identity mapping. In this way, theoretically, the network is in an optimal state, and the performance of the network will not decrease as the depth increases. Each layer of ResNet 101 will respond to or activate the input image. However, only a few layers in ResNet 101 are suitable for image feature extraction. The layer at the beginning of the network captures basic image features, such as edges and spots. Figure 26 shows the visualization of the network filter weights from the first convolutional layer. The first layer has 64 separate weight sets.

Next, the ResNet101 parameter setting is explained. The algorithm is specified as “sgdm”, which uses the Stochastic Gradient Descent (SGDM) optimizer with momentum. The settings are as follows: Verbose is 0, VerboseFrequency is 30, MaxEpochs is 6, MiniBatchSize is 2, ValidationFrequency is 6, ValidationPatience is 6, InitialLearnRate is 0.000100, LearnRateSchedule is 0, LearnRateDropPeriod is 10, LearnRateDropFactor is 0.10. The learning rate is an important hyperparameter in deep learning. A proper learning rate can make the loss function converge to a local minimum in a short time. Setting the learning rate too low will result in slower network convergence and longer training time, and too large a setting may cause the gradient to oscillate near the minimum or even fail to converge. L2Regularization is 0.0000500, Momentum is 0.96, GradientThreshold is Inf, GradientThresholdMethod is l2norm, the SequenceLength is the longest, SequencePaddingValue is 0, and ExecutionEnvironment is gpu. Figure 26 shows the classification result of ResNet101, and the accuracy rate is 94%. In the six categories, the classification accuracy rate is 100% for imbalance, normal, and underhang bearing, and for horizontal misalignment, the classification accuracy rate is 82.4%. The error classification is 1 each for overhang bearing, underhang bearing, and vertical misalignment. The error rate is 17.6%, and the classification accuracy rate for overhang bearing is 93.3%. When misclassified to 1 for underhang bearing, the error is 6.7%, the vertical misalignment classification accuracy rate is 93.3%, and for misclassification of 1 for horizontal misalignment, the error is 6.7%. After a comprehensive evaluation, the accuracy of AlexNet’s classification result is 75%, and the accuracy of GoogLeNet’s classification result is 85.7%. The accuracy of ResNet101′s classification result is 94%. Therefore, after the VMD analysis was completed, ResNet101 was shown to have the highest classification prediction accuracy rate.

## 5. Conclusions

With the rapid development of science and technology and industrial Internet of Things technology, mechanical equipment has shown the characteristics of being large-scale, complex, and intelligent, and large amounts of data are produced. In the actual operation process, because motor equipment often works under variable working conditions in which the speed and load are not constant, coupled with the mutual correlation and close coupling between the components, the non-linear and non-stationary characteristics of the vibration signal are significant. Traditional fault diagnosis methods based on constant working conditions and stationary signals are prone to the misdiagnosis or missed diagnosis of faults. Intelligent diagnosis of motor faults is an important means to ensure the safe operation of equipment driven by data. To accurately identify the health status of equipment, intelligent diagnosis needs to rely on enough available monitoring data to train an intelligent diagnosis model. The conclusions of this research are explained as follows. Our work summarizes the domestic and foreign research progress and development trends of motor intelligent fault diagnosis points out the challenges of the theory and methods of motor intelligent fault diagnosis in the context of big data, and finally discusses the solutions and development trends to deal with these challenges. The contribution of the research results is the proposal of an automatic fault diagnosis system combining VMD and ResNet101. First, the VMD method is used to analyze the motor vibration signal, and the obtained IMF components are used. Then, a Hilbert transform is performed on each IMF component, and the Hilbert marginal spectrum is obtained. Predictions are classified through ResNet101, GoogLeNet, and AlexNet in deep learning methods. The data verification is divided into the training set, and test set samples, and a fault diagnosis model based on the VMD–ResNet101 method is established. Finally, the deep neural network built by training and testing can determine the accuracy of diagnosis. ResNet101 has an accuracy rate of 94%, GoogLeNet has an accuracy rate of 85.7%, and AlexNet has an accuracy rate of 75%. The proposed method does not require a large amount of prior knowledge of fault diagnosis, does not need to denoise the signal, simplifies the feature extraction process of motor fault diagnosis, and has a high fault diagnosis accuracy rate. This research method can effectively identify the health of a motor.

## Figures and Tables

**Table 1:** Experimental equipment specifications.

Specification	Value	Unit
Motor	1/4	CV DC
Frequency range	700–3600	rpm
System weight	22	kg
Axis diameter	16	mm
Axis length	520	mm
Rotor	15.24	cm
Bearing distance	390	mm

**Table 2:** Experiments on motor failure conditions and numbers of failures.

Sequence	Number
Normal (no fault)	48
Horizontal misalignment	49
Vertical misalignment	50
Imbalance	48
Underhang bearing	37
Overhang bearing	49

**Table 3:** The network structures of the three methods.

Method	Number of Network	Type	Size	Parameters (Millions)	Image Input Size
AlexNet	25	Series	227 MB	61.0	227×227×3
GoogLeNet	22	DAG	27 MB	7.0	224×224×3
ResNet101	101	DAG	167 MB	44.6	224×224×3

**Table 4:** Comparison of the parameters of the three methods.

	ResNet101	GoogLeNet	AlexNet
Training Option			
Solver name (algorithm)	sgdm	sgdm	sgdm
Mini-Batch Options			
MaxEpochs	6	6	6
MiniBatchSize	2	2	2
Shuffle	Every epoch	Every epoch	Every epoch
Validation			
ValidationFrequency	6	6	6
ValidationPatience	6	6	6
Solver Options			
InitialLearnRate	0.000100	0.000100	0.000100
Learn-RateSchedule	none	none	none
MiniBatchSize	10	10	10
LearnRateDropFactor	0.10	0.10	0.10
L2Regularization	0.00005	0.00005	0.00005
Momentum	0.96	0.96	0.96
Gradient Clipping			
GradientThreshold	Inf	Inf	Inf
GradientThresholdMethod	l2norm	l2norm	l2norm
Sequence Options			
SequenceLength	longest	longest	longest
SequencePaddingValue	0	0	0
Hardware Option			
ExecutionEnvironment	GPU	GPU	GPU

**Table 5:** Comparison of the characteristics of the three methods.

	ResNet101	GoogLeNet	AlexNet
Characteristics	The input data of the residual block are subjected to identity mapping through a shortcut connection. Basicblock and Bottleneck structures are used to upgrade and reduce the number of channels (cross-channel information integration).	The pure convolutional layer and pooling layer are changed to Inception architecture. Average pooling is used to replace the fully connected layer in the final classification. The network added 2 auxiliary classifiers in order to avoid the disappearance of the gradient.	A non-linear activation function (activation function) is used as ReLU. Dropout and data augmentation are used to reduce overfitting.

## Data Availability

Not applicable.

## References

[1] Murphey Y.L., Masrur M., Chen Z., Zhang B. (2006). Model-based fault diagnosis in electric drives using machine learning. IEEE/ASME Trans. Mechatron..

[2] Kankar P., Sharma S.C., Harsha S. (2011). Fault diagnosis of ball bearings using machine learning methods. Expert Syst. Appl..

[3] Tashakori A., Ektesabi M.M. Fault diagnosis of in-wheel BLDC motor drive for electric vehicle application. Proceedings of the 2013 IEEE Intelligent Vehicles Symposium (IV).

[4] Praveenkumar T., Saimurugan M., Krishnakumar P., Ramachandran K. (2014). Fault diagnosis of automobile gearbox based on machine learning techniques. Procedia Eng..

[5] Ulatowski A., Bazzi A.M. (2015). A combinational-logic method for electric vehicle drivetrain fault diagnosis. IEEE Trans. Ind. Appl..

[6] Vakharia V., Gupta V.K., Kankar P.K. (2015). Ball bearing fault diagnosis using supervised and unsupervised machine learning methods. Int. J. Acoust. Vib..

[7] Ma C., Liu Q., Wang D., Li Q., Wang L. (2015). A novel black and white box method for diagnosis and reduction of abnormal noise of hub permanent-magnet synchronous motors for electric vehicles. IEEE Trans. Ind. Electron..

[8] Xu X., Wang H., Zhang N., Liu Z., Wang X. (2017). Review of the fault mechanism and diagnostic techniques for the range extender hybrid electric vehicle. IEEE Access.

[9] Zhou H., Liu Z., Yang X. (2017). Motor Torque Fault Diagnosis for four wheel independent motor-drive vehicle based on unscented kalman filter. IEEE Trans. Veh. Technol..

[10] Qi G., Zhu Z., Erqinhu K., Chen Y., Chai Y., Sun J. (2018). Fault-diagnosis for reciprocating compressors using big data and machine learning. Simul. Model. Pract. Theory.

[11] Ali M.Z., Shabbir N.S.K., Liang X., Zhang Y., Hu T. (2019). Machine learning-based fault diagnosis for single- and multi-faults in induction motors using measured stator currents and vibration signals. IEEE Trans. Ind. Appl..

[12] Hu C., Tang X., Zou L., Yang K., Li Y., Zheng L. (2019). numerical and experimental investigations of noise and vibration characteristics for a dual-motor hybrid electric vehicle. IEEE Access.

[13] Huang H.B., Huang X.R., Wu J.H., Yang M.L., Ding W.P. (2019). Novel method for identifying and diagnosing electric vehicle shock absorber squeak noise based on a DNN. Mech. Syst. Signal Process..

[14] Huang G., Luo Y.-P., Zhang C.-F., Huang Y.-S., Zhao K.-H. (2015). Current Sensor Fault Diagnosis Based on a Sliding Mode Observer for PMSM Driven Systems. Sensors.

[15] Chang H.-C., Jheng Y.-M., Kuo C.-C., Hsueh Y.-M. (2019). Induction Motors Condition Monitoring System with Fault Diagnosis Using a Hybrid Approach. Energies.

[16] Jeon N., Lee H. (2016). Integrated Fault Diagnosis Algorithm for Motor Sensors of In-Wheel Independent Drive Electric Vehicles. Sensors.

[17] Jing L., Wang T., Zhao M., Wang P. (2017). An Adaptive Multi-Sensor Data Fusion Method Based on Deep Convolutional Neural Networks for Fault Diagnosis of Planetary Gearbox. Sensors.

[18] Hsueh Y.-M., Ittangihal V.R., Wu W.-B., Chang H.-C., Kuo C.-C. (2019). Fault Diagnosis System for Induction Motors by CNN Using Empirical Wavelet Transform. Symmetry.

[19] Xue H., Zhou J., Wang M., Li Z., Jiang H. (2019). Using rotating speed monitoring for leakage fault diagnosis of in-wheel motor. J. Appl. Sci. Eng..

[20] Li S., Liu G., Tang X., Lu J., Hu J. (2017). An Ensemble Deep Convolutional Neural Network Model with Improved D-S Evidence Fusion for Bearing Fault Diagnosis. Sensors.

[21] Goyal D., Dhami S.S., Pabla B.S. (2020). Non-Contact fault diagnosis of bearings in machine learning environment. IEEE Sens. J..

[22] He C., Wu T., Liu C., Chen T. (2020). A novel method of composite multiscale weighted permutation entropy and machine learning for fault complex system fault diagnosis. Measurement.

[23] Meckel S., Schuessler T., Jaisawal P.K., Yang J.-U., Obermaisser R. (2020). Generation of a diagnosis model for hybrid-electric vehicles using machine learning. Microprocess. Microsyst..

[24] Chang L.-K., Wang S.-H., Tsai M.-C. (2020). Demagnetization Fault Diagnosis of a PMSM Using Auto-Encoder and K-Means Clustering. Energies.

[25] Gundewar S.K., Kane P.V. (2021). Condition Monitoring and Fault Diagnosis of Induction Motor. J. Vib. Eng. Technol..

[26] Xiao F. (2017). A Novel Evidence Theory and Fuzzy Preference Approach-Based Multi-Sensor Data Fusion Technique for Fault Diagnosis. Sensors.

[27] Tra V., Kim J., Khan S.A., Kim J.-M. (2017). Bearing Fault Diagnosis under Variable Speed Using Convolutional Neural Networks and the Stochastic Diagonal Levenberg-Marquardt Algorithm. Sensors.

[28] Hua X., Thomas A., Shultis K. (2021). Recent progress in battery electric vehicle noise, vibration, and harshness. Sci. Prog..

[29] Rauber T.W., Loca A.L.D.S., Boldt F.D.A., Rodrigues A.L., Varejão F.M. (2021). An experimental methodology to evaluate machine learning methods for fault diagnosis based on vibration signals. Expert Syst. Appl..

[30] Toma R.N., Prosvirin A.E., Kim J.-M. (2020). Bearing Fault Diagnosis of Induction Motors Using a Genetic Algorithm and Machine Learning Classifiers. Sensors.

[31] Xiao D., Qin C., Yu H., Huang Y., Liu C. (2021). Unsupervised deep representation learning for motor fault diagnosis by mutual information maximization. J. Intell. Manuf..

[32] Xue H., Wu M., Zhang Z., Wang H. (2021). Intelligent diagnosis of mechanical faults of in-wheel motor based on improved artificial hydrocarbon networks. ISA Trans..

[33] Huang N.E., Shen Z., Long S.R., Wu M.C., Shih H.H., Zheng Q., Yen N.-C., Tung C.C., Liu H.H. (1998). The empirical mode decomposition and the Hilbert spectrum for nonlinear and non-stationary time series analysis. Proceedings of the Royal Society A: Mathematical. Phys. Eng. Sci..

[34] Dragomiretskiy K., Zosso D. (2013). Variational mode decomposition. IEEE Trans. Signal Process..

[35] Lawrence S., Giles C.L., Tsoi A.C., Back A.D. (1997). Face recognition: A convolutional neural-network approach. IEEE Trans. Neural Netw..

[36] Mikolov T., Karafiát M., Burget L., Černocký J., Khudanpur S. Recurrent neural network based language model. Proceedings of the Eleventh Annual Conference of the International Speech Communication Association.

[37] He K., Zhang X., Ren S., Sun J. (2016). Identity mappings in deep residual networks. arXiv.

[38] Krizhevsky A., Sutskever I., Hinton G.E. (2012). Imagenet classification with deep convolutional neural networks. Adv. Neural Inf. Process. Syst..

[39] Szegedy C., Liu W., Jia Y., Sermanet P., Reed S., Anguelov D., Erhan D., Vanhoucke V., Rabinovich A. Going deeper with convolutions. Proceedings of the IEEE Conference on Computer Vision and Pattern Recognition.

